# Onboarding in Polish Enterprises in the Perspective of HR Specialists

**DOI:** 10.3390/ijerph20021512

**Published:** 2023-01-13

**Authors:** Agnieszka Krugiełka, Grażyna Bartkowiak, Agnieszka Knap-Stefaniuk, Ewa Sowa-Behtane, Ryszard Dachowski

**Affiliations:** 1 Faculty of Engineering Management, Poznan University of Technology, Jacka Rychlewskiego 2, 60-965 Poznan, Poland; 2 Faculty of Humanities and Social Sciences, Naval Academy in Gdynia, ul. Śmidowicza 69, 81-127 Gdynia, Poland; 3 Faculty of Education, Institute of Political and Administrative Sciences, Jesuit University Ignatianum in Krakow, ul. Kopernika 26, 31-501 Kraków, Poland; 4 Faculty of Education, Institute of Educational Sciences, Jesuit University Ignatianum in Krakow, ul. Kopernika 26, 31-501 Kraków, Poland; 5 Faculty of Civil Engineering and Architecture, Kielce University of Technology, al. 1000-Lecia PP 7, 25-314 Kielce, Poland

**Keywords:** sustainable development, onboarding, phases of onboarding, components of onboarding, types of onboarding, HR specialists, employees

## Abstract

The article discusses onboarding in Polish enterprises analyzed from the perspective of HR specialists. The subject of consideration in the article falls within the area of broadly understood concern for the sustainable development and well-being of employees adjusting to a new work environment during their adaptation period. Actions taken by HR specialists have a significant impact on the behavior of employees and their well-being and satisfaction in the new workplace, including commitment and care for all stakeholders involved in building a positive psychological climate at the time of starting work. The aim of the article was to identify onboarding practices implemented in selected types of Polish enterprises and selected factors determining the choice of specific practices, such as analyzing the dependencies between the type of an enterprise and the implementation of onboarding practices in it, identifying the perception of these practices by HR male and female specialists at a given age and with given seniority, and determining whether the practice of assigning a buddy to new employees depends on the type of enterprise. The study described in the article was conducted in 178 medium-sized Polish enterprises, of which, 25 were manufacturing companies, 34 were trading companies, and 119 were service companies. In each enterprise, an HR specialist completed a questionnaire on onboarding. The analysis of the dependencies between the type of company and the type of onboarding implemented in it (general, position, or team onboarding) revealed no statistically significant differences between the types of companies participating in the study and the type of onboarding implemented in them. The discrepancies between preferences for particular onboarding practices and the gender, age, and seniority of HR specialists participating in the study turned out to be statistically significant and indicate that manufacturing companies more often assign a buddy to new employees in their onboarding process than trading and service companies. The conclusions obtained from the research, apart from the cognitive value, have an application value, e.g., regarding the recruitment of HR specialists.

## 1. Introduction

The subject matter of our article was prepared within the paradigm of sustainable development, the understanding of which has recently expanded significantly. It includes not only care for the preservation of resources related to the environment, counteracting the pollution of the environment, taking action against global warming, and the transformation of energy systems, but also care for the functioning of organizations, enterprises, and the existence of certain procedures related to the personnel policy; in particular, to onboarding, the existence of which influences the well-being of employees regardless of age, gender, state, health, etc. [1,2,3,4,5].

Care for the employee at the moment of taking up employment as an HR function is a significant element of the general care for working conditions, the functioning of a wide social group, and the prospects for professional development.

In turn, actions taken by HR specialists determine the behavior of employees and their well-being and satisfaction in the new workplace, including commitment and care for all stakeholders involved in building the broadly treated sustainable development of the organization. Onboarding serves people, constitutes an important element of the psychological contract [6], and allows a person to be better adapted to the new work environment.

Organizations and researchers have been searching for meaningful and positive relations between practices implemented within employee well-being [7], human resource management [8,9,10,11,12], employee attitudes, values, employee productivity [13,14,15,16,17], and company performance [18,19,20,21] for over two decades. Analyzing current tendencies, it can be concluded that the concept of universal good practices is losing relevance and is being replaced by personalized HR practices, as research findings reveal that practices that work best in one company do not necessarily have the same impact and influence in other companies. Thus, it can be concluded that “one good practice does not fit all companies” and that a diversification of activities is currently taking place, both in relation to the size of companies, their sector, and the specificity of their operations [21] and country [22,23]. 

Present and future HR priorities are linked to attracting talent as a way to ensure that organizations possess the right key competencies. The main objectives of enterprises include attracting and retaining the right people by offering them performance-related rewards and competency development programs, including training, as well as reducing job turnover and offering mentoring, coaching, and shadowing practices [24,25,26]. While theoretical studies still focus on recruiting [27,28], developing [29,30], and retaining a talented workforce [31,32], especially young university graduates [33,34], it seems that no other element of human capital management has so far been as neglected by companies as onboarding: introducing new employees to their new work environment. 

The implementation of onboarding practices cannot simply be reduced to formal one- or two-day conference room sessions, during which, someone from the HR department briefs the newcomers on a company’s structure, mission and goals, and internal policies and code of ethics [35]. Onboarding should also include a wide range of socialization activities directed at new employees [36], and it should take place in a processual manner and in a way that is adapted to the specifics of a given enterprise [37,38,39]. Sometimes, as their onboarding practice, companies assign a buddy to their new employees, whose task is to take care of them. Furthermore, effective onboarding should consider individual adaptation factors (or determinants) of a future employee [40,41,42,43,44,45]. 

Despite the fact that the issue of onboarding is being addressed more and more often, the literature on the subject, which focuses more on theoretical considerations, lacks sufficiently diverse empirical research that allows for the implementation of their application results into management practice. The article prepared by the authors aims to fill the perceived gap.

The aim of the article is to identify onboarding practices as a significant element of HRM implemented in selected types of Polish enterprises, and to analyze relationships between the type of an enterprise and the way in which new employees are introduced to their new workplace. Moreover, the article seeks to find out the opinions of HR specialists of a given gender, age, and seniority on these practices and to answer the question of whether the practice of assigning a buddy to new employees depends on the type of an enterprise.

## 2. Theoretical Considerations

### 2.1. The Concept of Onboarding

The purpose of this part of the article is to describe onboarding and to familiarize the reader with knowledge about the dynamics of the development of the term onboarding.

The origins of onboarding can be traced back to the mid-19th century, when it was noticed that an extremely important aspect in an organization was the potential of the employees’ talent, regardless of their position in the organizational structure. 

A significant impact of onboarding, and, at the same time, its cause, was the trend within companies’ development to introduce employee development programs. At the end of the previous century, some employers struggled with a shortage of qualified managerial staff. At that time, the war for talent (which had been mentioned by specialists almost 150 years earlier) began [46]. 

The definition formulated fifteen years ago, proposed by Frear [47], treats onboarding from a holistic perspective, taking into account people, processes, and technology. Later, a greater focus was placed on specific practices implemented during the recruitment of an employee to work and their subsequent analysis. One of the different approaches to onboarding was related to practices taken by companies by the development of new solutions in the onboarding process as an expression of their concern for new employees.

In the most frequently quoted approach to onboarding (formulated by Bauer), this term refers to the process of helping new employees to adjust to social and performance aspects of their new workplaces [40]. An older definition, proposed by Frear [47], can also be found in the literature, which approaches onboarding from a holistic perspective that takes into consideration people, processes, and technology. Combining these two definitions emphasizes the impact of employees’ productivity on the performance of an entire organization. Importantly, these employees should be properly introduced to their new work environment [36,46,48].

Onboarding is a highly relevant issue, as evidenced by numerous recent publications of researchers from various countries and various scientific disciplines, including management [49,50,51,52,53,54], psychology, education, and others [55,56,57,58,59].

When describing an onboarding process, some authors distinguish between the orientation and socialization of new employees [60,61,62,63]. Orientation, as defined by Wanous [64,65,66], involves the implementation of short-term programs that provide new employees with basic information about their duties (employment documents and skills training) in order to reduce stress related to starting a new job. Through the implementation of specific activities, practices, or programs, organizations can eliminate a number of negative phenomena, e.g., task ambiguity, which triggers conflict and other stressful situations, and thus contributes to new employees’ satisfaction with their work and their being accepted by co-workers [67]. Ellice [68] emphasizes that, from the perspective of an organization, this stage is an extensive process of preparation, integration, and other follow-up activities directed at the socialization of new employees. A successful socialization process means that new employees internalize values, norms, and the mission and vision of an organization [69]. 

In the most up-to-date approach to onboarding, according to Klein and Polin [70], onboarding refers to specific practices implemented by an organization in order to help employees adjust to their new roles in line with an employer’s expectations that onboarding, as a process implemented in an organization that hires new employees positively, correlates with their job satisfaction, organizational attachment, and engagement and productivity, and reduces employee turnover (e.g., Klein and Weaver, [71]; Lavigna, [72]). Furthermore, bearing in mind that, in 2015, the average cost of recruiting one employee in Poland amounted to over PLN 2500 [73] and that approximately 17% of new employees give up their jobs soon after being employed, onboarding can certainly lead to a lower turnover rate in companies. 

Summing up, onboarding can currently be defined as particular measures undertaken by a company that allow new employees to best adjust to their new workplace in such a way that they can use their potential (knowledge, competences, experience, etc.) to the fullest extent possible, reduce the level of stress associated with change (cf. Holmes, Rahe) [74], and, at the same time, contribute effectively to the success of an organization. Onboarding, understood as a process of introducing an employee to work, is implemented in several phases, consists of particular components, and performs specific functions.

It should be emphasized that onboarding is currently part of the sustainable development paradigm, the understanding of which has recently been broad and also applies to HRM practices. Sustainable development includes the functioning of enterprises and, as part of sustainable HRM, the existence and implementation of solutions related to personnel policy that significantly affect the well-being of employees in organizations.

### 2.2. Levels, Components, and Types of Onboarding

Bauer [50] identified four levels of employees’ adjustment to a company, related to legal matters, efficiency (linked to productivity required), adaptation to organizational culture, and relationships with other employees, which she called, respectively, compliance, clarification, culture, and connection. The last of them, the level of compliance, focuses on ensuring new employees’ compliance with all formal and legal requirements in accordance with existing procedures and customs, e.g., the way they dress, address others, etc. According to Meyer and Bartels [58], activities undertaken at this level should also include employees’ familiarization with a special guide prepared by a company for new employees. During the level of clarification, employees should understand what an organization expects of them if they are to meet adopted standards, which requires that they confront their competences. The culture level introduces employees to the history of their company’s operations and its norms and values. It requires employees’ acceptance of its philosophy [75]. The connection level is linked with establishing formal and informal relationships within an organization and is the culmination of the previous levels. When planning its onboarding process, an organization should account for all of these four levels in order to help new employees to successfully settle in.

However, not all authors approach the concept of onboarding in such a model. Cesario and Chambel [53] used the notion of a strategic onboarding model, which consists of three components: structured corporate welcome, manager welcome, and co-workers welcome. Although this understanding of onboarding is in line with Holton’s [69] strategic approach, it seems relatively narrow, as it covers only the initial phase of onboarding. 

From the employees’ perspective, an effective onboarding process contributes to their faster adaptation to a company’s culture and goals, to reducing their uncertainty and anxiety related to not meeting the company’s performance expectations, to facilitating their adjustment to a new work environment, and, generally, to their acquisition of desired or necessary attitudes, behaviors, and knowledge [76,77]. An employer also benefits in many ways from onboarding new employees into their new workplace. 

In the analyzed literature, the authors did not find articles referring to the results of research on onboarding carried out among HR specialists, and they are particularly responsible for specific solutions used in companies as part of the personnel policy of a given company. The research described in this article can therefore be treated as a basis for more in-depth analyses of onboarding from the perspective of HR specialists.

The analysis of the literature cited in the article allowed for distinguishing the following types of onboarding: general onboarding, position onboarding, and team onboarding. *General onboarding* consists of completing all formalities required by law and providing new employees with basic information about an organization. During *position onboarding*, employees are introduced to the departments or organizational units that are the most important from the point of view of their position, as well as to their duties and the ways of performing them. *Team onboarding* covers activities related to handing employee’s necessary equipment and familiarizing them with the configuration and setup of the tools used at work, the mode of their work, and members of their teams. 

In the next part, unique indicators will be assigned to each type of onboarding with a view to identifying the type of onboarding. Onboarding practices include particular activities undertaken by companies, such as videos posted on the Internet/intranet, presentations, leaflets, and face-to-face meetings. 

Based on the aforementioned criteria, the authors distinguished one more onboarding practice, namely assigning a buddy to a new employee. This practice seems to contain all of the elements of the types of onboarding identified, and thus can be preferred by HR specialists.

## 3. Methodology

### 3.1. Research Area

The research methodology will be discussed in the following order: assumptions justifying the research issues, research goals and problems, and research tools.

The article assumes that onboarding in modern enterprises is an element of HRM. In turn, HRM is part of the broadly understood concept of sustainable development. Therefore, the analysis of onboarding solutions from the perspective of HR specialists is an important element in the discussion on various aspects of sustainable development in relation to the functioning of modern enterprises and the solutions used by them in relation to their employees.

The aim of the article was to diagnose and analyze the implementation of the onboarding process in selected types of Polish enterprises, to analyze the preferences of HR specialists for particular types of onboarding, depending on their gender, age, and seniority, and to determine whether the practice of assigning a buddy to a new employee as part of the onboarding process depends on the type of company. In order to achieve this aim, the following research problems were formulated:

1. What type of onboarding is most common in different types of Polish enterprises?
2. Does the preference for particular onboarding practices depend on the gender, age, and seniority of an HR specialist who prepares and implements the onboarding process in an enterprise?
3. Does the introduction of a buddy practice depend on the type of enterprise?

### 3.2. Research Tool

The procedure of designing the research tool was based on the methodology developed by Wąsek [78,79] and began with collecting and analyzing onboarding practices to be implemented if a new employee is to reach a desired level of adjustment and productivity in performing work in a new workplace The practices included in the research tool were selected on the basis of both the literature and the opinions of HR specialists whose companies implement programs related to the onboarding of new employees. 

This part of the development of the research procedure was exploratory in nature; therefore, the research procedure used can be classified as mixed procedures. The respondents participated in an interview, which included detailed questions about the type of practices and how they were implemented.

The first stage of designing the questionnaire involved identifying a set of practices, which were next analyzed by expert judges (expert judges were four specialists, two theoreticians, and two practitioners, with more than ten years of work experience in the HR field). As a result of this analysis, ten practices were chosen and assigned to one of the three types of onboarding identified earlier and used in designing the questionnaire. The order of individual items in the questionnaire resulted from the order of identification of the established types of onboarding.

Practices within general onboarding:

- A referral to pre-employment medical examination.
- Handing tools/equipment necessary for performing the job (e.g., laptop, phone, clothing).
- Granting access to places and services necessary for performing the job (e.g., parking lot, employee ID card, email address).
- Providing information on the company’s values, vision, and mission.
- Ensuring that employee hiring formalities are completed properly.
- Providing information on the company’s policies, procedures, and regulations.

Practices within position onboarding:

- Providing information on the departments or organizational units that are the most important from the point of view of an employee’s position.
- Providing information on an employee’s duties and ways of performing them.

Practices within team onboarding:

- Introducing employees to their superiors.
- Introducing employees to their department/team.

The above onboarding practices were used in designing the research tool—the questionnaire with 17 questions—which also addressed issues related to other HR practices. Seven initial questions allowed for the identification of the type of enterprise, its industry, and demographic data of people participating in the research. Each onboarding practice was assigned one point. The reliability of twelve multi-part questions regarding the implementation of the onboarding process was edited according to the Likert scale assumptions (this reliability was calculated with the Cronbach’s alpha and equaled 0.78).

## 4. Results

The analysis of the research results will include company-type presentations, characteristics of the respondents participating in the research—HR specialists—and an analysis of the research results in accordance with the formulated research problems.

### 4.1. The Type of Company (in Which an HR Specialist Works) and the Type of Onboarding Implemented

The analysis of the dependencies between the type of a company and the type of onboarding implemented in it (general, position, or team onboarding) did not reveal statistically significant differences between the types of companies participating in the study in which the respondents (HR specialists) work and the type of onboarding implemented in them. 

Table 1 shows the results (arithmetic means and standard deviations) related to the occurrence of practices associated with a specific type of onboarding in particular types of companies. This means that the companies analyzed in the study, regardless of their type, implement relatively balanced and comprehensive onboarding, understood as a set of various practices, thus demonstrating their concern for new employees. As can be seen from the table above, the companies implement different types of onboarding to a similar extent.

### 4.2. Characteristics of the Respondents

**Figure 1:**
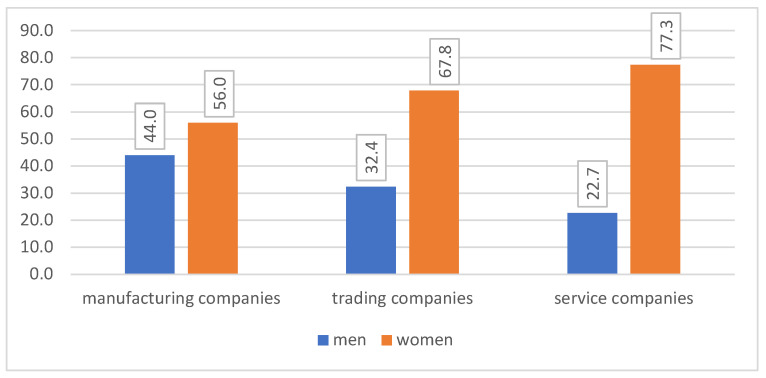
Respondents’ gender (percentage) in different types of companies. Source: own elaboration based on the results of the study.

The study was conducted in 178 medium-sized Polish companies, including 25 manufacturing companies, 34 trading companies, and 119 service companies. The questionnaire was completed by an HR specialist from each company. The selection of both the companies and HR specialist that took part in the study was based on purposive sampling. The condition for participation in the study was the agreement of the company’s management and HR specialist, and this participation was voluntary (at each stage, the respondents could opt out of completing the research tool). Detailed characteristics of the respondents are provided below in Figure 1. The study was conducted online over a one-and-a-half-year period between 2020 and 2022. All of the respondents held a degree (BA, MA, or Beng).

**Figure 2:**
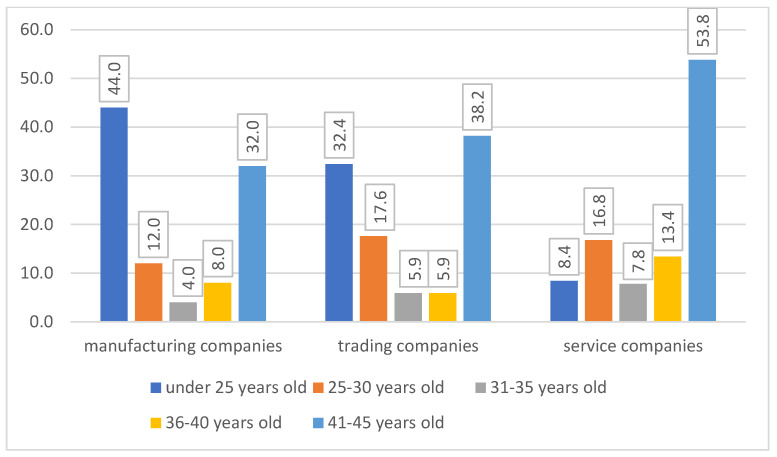
Respondents’ age (percentage) in different types of companies. Source: own elaboration based on the results of the study.

More women participated in the study than men (in each type of a company). Among the respondents from manufacturing companies, 44% were men and 56% were women; among the respondents from trading companies, 32% were women and 68% were men; and in service companies, 23% of the respondents were men and 77% were women, Figure 2.

Participation in the study of people with the above-mentioned, limited age categories resulted from the relatively low age of HR specialists. The largest number of HR specialists who participated in the study was in the age range between 41 and 45 (54% of the respondents from service companies, 38% of respondents from trading companies, and 23% of the respondents from manufacturing companies).

**Figure 3:**
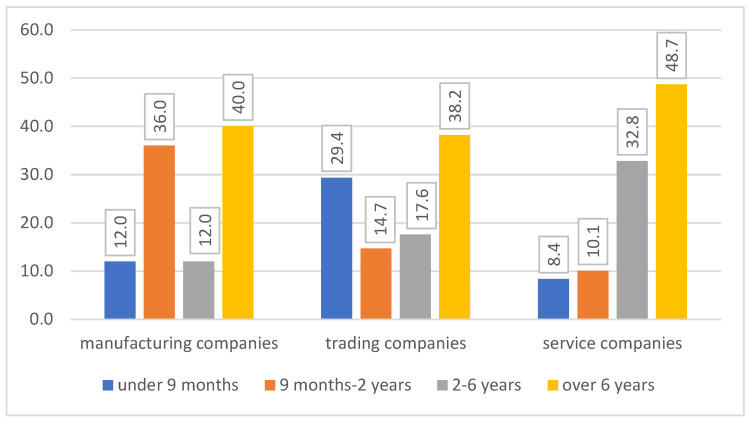
Respondents’ seniority (percentage) in different types of companies. Source: own elaboration based on the results of the study.

The selection of people with different seniority (Figure 3) for the study resulted due to two reasons: significant discrepancies regarding the functioning of people with different seniority in the company (cf. Krugiełka, 2019, p. 133–134) and because of the realistic opportunities of conducting research. Participation in the research was voluntary, so it did not exhaust all HR specialists employed in the companies taking part in the research.

The largest number of HR specialists who participated in the study was from those with seniority of more than six years (40% of the respondents from manufacturing companies; 39% of the respondents from trading companies; and 49% of the respondents from service companies).

### 4.3. Preference for Particular Onboarding Practices and Respondents’ Gender

The analysis of the results reveals the respondents’ preference for a particular practice in the onboarding process depending on their gender. The table below shows the results regarding the respondents’ preferences for a particular type of onboarding.

As can be seen from Table 2, the results indicate that onboarding practices chosen by the respondents are comparable for female and male HR specialists. However, it should be emphasized that female HR specialists attach more importance than men to granting new employees’ access to places and services necessary in their new workplace (parking lots, ID employee cards, e-mail addresses, etc.). The discrepancy between the preferences for implementing the practice of general onboarding related to granting access is statistically significant (*p* = 0.17, Table 3). Perhaps this preference stems from women’s more pragmatic approach to the realities of everyday life, as they usually have to combine their family and work responsibilities. Hence, in the practice of general onboarding, giving new employees everything that will facilitate their successful functioning in their new work environment is more important for women.

### 4.4. Preference for Particular Onboarding Practices and the Respondents’ Age

Another aspect analyzed in the study was whether the age of HR specialists (independent variable) is a grouping variable for the implementation of particular practices in different types of companies (dependent variable). 

As the data in Table 2 indicate, there is a statistically significant difference between different age groups as a differentiating factor in the implementation of the practice related to providing new employees with information on the company’s values, vision, and mission. The results obtained reveal a statistically significant difference (*p* = 0.39) between the respondents’ age (belonging to a given age group) and their preference in providing new employees with relevant knowledge about the company. This means that age is both a grouping variable and an independent variable in the area of preference for the practice related to providing new employees with information on the company’s values, vision, and mission. Depending on their age, HR specialists varied in their assessments of how important it is for a new employee to be informed about different aspects of the company’s culture (listed in Table 2) and how this culture operates in practice. Perhaps older employees have a greater appreciation of the importance of knowing the company’s values, mission, and vision for its employees. This attitude might stem from their experience, which allows them to appreciate the role of the compatibility of employees’ professed values with those of the organization as a factor in ensuring the effective functioning of new employees in an organization.

Table 4 and Table 5 present the results regarding the preferences for particular onboarding practices among the respondents at a given age range. These preferences cover the practices implemented within three types of onboarding: general, team, and position.

### 4.5. Preference for Particular Onboarding Practices and the Respondents’ Work Experience (Seniority)

Considering the respondents’ seniority as a criterion for grouping their answers (independent variable), the results obtained revealed differences in their recognition of the importance of the practice related to handing tools/equipment necessary for performing the job (e.g., laptop. phone, appropriate clothing). This means that, depending on their seniority, HR specialists attach different importance to informing new employees about the tools that facilitate their adjustment to a new workplace. Table 6 and Table 7 present the preferences for a particular type of onboarding among HR specialists with given seniority. 

The statistical analysis revealed a statistically significant difference (*p* = 0.035) granting access to places and services necessary for performing the job (e.g., parking lot, employee ID card, email address). It can be assumed that this practice of general onboarding, in the opinion of the respondents, considerably facilitates the functioning of new employees in the workplace. From the analysis of practices of team onboarding, seniority-dependent differences were also observed between employees with work experience between 2–6 years and those with 9 months-2 years in the area of introducing employees to their team. 

This result indicates that preferences related to new employees’ need to establish relationships with their co-workers as a significant element of the onboarding process differ depending on the HR specialists’ seniority. The opportunity to establish these relationships allows new employees to directly ask for information needed to perform their job, which positively affects their well-being, reduces the uncertainty related to their new workplace, and builds their sense of security.

### 4.6. The Type of Company and Assignment of a Buddy

The analysis of the relationship between a company’s assignment of a buddy to new employees and the type of company revealed a statistically significant relationship between these variables, Table 8.

This relationship indicates that manufacturing companies whose HR specialists participated in the study more often assign a buddy to new employees during the onboarding process than trading and service companies and that this difference is statistically significant (*p* = 0.16; Table 9).

It can be assumed that this result is linked with the expectations of new employees stemming from the specificity of work in manufacturing companies, which requires specific knowledge and qualifications as well as direct contact with more experienced employees who are already familiar with the specificity of work in a given company, which is the most effective way to obtain necessary information and dispel possible doubts at a particular position in a new workplace.

### 4.7. Summary 

As the results obtained in the study demonstrate, there is no correlation between the type of onboarding (general, team, or position onboarding) implemented in companies and the type of company (manufacturing, trading, or service company). Perhaps, an important role in the implementation of the onboarding process is played less by the size of an enterprise and more by its financial condition, how long it has been in the market (its onboarding practice and experience), or the level of its technological development. However, the results do reveal different preferences of the respondents of different gender, age, and seniority related to the type of onboarding. 

Female HR specialists and employees with a higher seniority find it more important than other groups of respondents to grant new employees access to places and services necessary for performing their jobs (e.g., parking lot, employee ID card, e-mail address), which facilitates the initial period of work in a new place. Not surprisingly, the respondents also appreciated introducing new employees to their teammates. The respondents’ confirmation of the vital role played by the socialization process in the onboarding phase seems valuable. This result not only highlights the importance of this factor as an element of the company’s organizational culture but also points to the need to consider possible difficulties in the socialization process and to apply appropriate preventive measures [78,79].

The last research problem analyzed in the study was related to the practice of assigning new employees a buddy whose task is to offer them support and to inform them about the requirements of their new job. As was already mentioned, it comes as a surprise that this practice is most frequently implemented in manufacturing companies (the differences between types of companies are statistically significant). However, it can be concluded that the implementation of this particular practice is related to the expectations of employees in such companies. Nevertheless, the authors are of the opinion that this conclusion cannot be generalized as manufacturing companies constituted only 14% of all the companies that participated in the study.

## 5. Discussion

The results of the study confirm the significant role of onboarding in the process of employees’ adjustment to new working conditions. Following Lavigna [72], the assumption that the implementation of the onboarding process is linked to an increased employee engagement and productivity and leads to a reduction in employee turnover leads to formulating a recommendation that companies should optimize their onboarding practices. The study revealed the involvement of different companies in this process, regardless of the type they represent. It is encouraging that all companies taking part in the study were involved in the socio-professional adaptation of new employees, both in terms of the type of onboarding and the concern to assign a buddy to new employees, which offers them a direct form of communication [80] and allows them to learn about their new workplace with relative ease. 

The results are also in line with those obtained by Lawong et al. [81], which emphasize the importance of a broadly understood recruitment process, including onboarding practices, for building a positive image of an organization. Building a positive image of an organization involves creating an effective employment brand (obtained through, among others, onboarding practices) that positively influences the performance at both organizational and personal levels, which further translates into a company’s competitive advantage. The resulting brand or commercial value can then be associated with a strong employer brand, which can also positively affect employee loyalty and reduce staff turnover [82]. Thus, the hiring process, especially the onboarding phase, is a category that strongly influences the perception of a company as an attractive workplace, especially for its current employees but also for potential future employees, who declare that the working environment, relationships within a company, management style, bonuses and benefits, and the type of work performed are important for them [83]. According to Backhaus and Tikoo [84], employee loyalty is a form of employees’ attachment to their employer and can also result from well-designed and professionally implemented onboarding practices. This attachment benefits an employer on various counts, increases the level of employees’ commitment to their work, contributes to increasing employees’ job satisfaction, and reduces turnover rates.

In a globalized world in which companies frequently operate in international markets, they face increasingly fierce competition on an everyday basis, and good HRM practices can prove crucial in providing an organization with a competitive advantage [85,86,87]. According to Gupta and Saxena [88], the current HRM philosophy/policy boils down to the implementation of a number of practices that aim to achieve a balance between striving to continually improve employees’ and an organization’s productivity and meeting employees’ needs, respecting their values, and increasing their well-being [89,90,91]. 

The study described in this article fits with such a vision of the functioning of contemporary organizations. When considering the applicability of the results obtained in the study, the authors believe that it is necessary to account for the age, seniority, position, and even gender of job candidates when planning the onboarding process and its particular practices. As the results demonstrate, these variables are relevant in the case of HR specialists, and are certainly particularly important in relation to new employees. However, it should be remembered that the dependencies found in the study need to be confirmed in further quantitative and qualitative studies (e.g., in-depth interviews). It is also advisable to diversify companies investigated in further studies, e.g., with regard to the previously mentioned criteria, such as their size, financial condition, how long they have been in the market, or level of their technological development.

## 6. Conclusions, Ethical Aspect of the Study, and Limitations and Directions for Further Research 

In the authors’ opinion, it should be emphasized that onboarding, understood as the process of introducing new employees to work, is successfully completed when an employee reaches at least an average degree of effectiveness in the following activities: adaptation to the system of work in a given company, which manifests in a significant decrease in fatigue; creativity in discussing and performing tasks assigned by a superior; full subordination to superiors (effective performance of duties and tasks assigned); being accepted by the immediate co-workers (developing positive relationships within a team); adaptation to the culture of a given company; satisfaction with one’s work, company, team, and co-workers.

The goal of the article was to identify onboarding practices implemented in selected types of Polish enterprises and to analyze relationships between the type of an enterprise and the way in which new employees are introduced to their new workplace. The article was aimed at getting to know the opinion of HR specialists of a given gender, age, and seniority on these practices and to answer the question of whether the practice of assigning a buddy to new employees depends on the type of enterprise.

The results of the study did not show a statistically significant relationship between the type of company and the type of onboarding implemented in it (general, position, or team onboarding), and revealed no statistically significant differences between the types of companies participating in the study and the type of onboarding implemented in them. The discrepancies between preferences for particular onboarding practices and the gender, age, and seniority of HR specialists participating in the study turned out to be statistically significant and indicate that manufacturing companies more often assign a buddy to new employees in their onboarding process than trading and service companies.

The results of the conducted research indicate, above all, that onboarding is used in various types of Polish enterprises, which proves that they care about newly hired employees in a new workplace for them. Research confirms that HR managers have knowledge about the solutions used in onboarding and apply them in practice. The age of HR specialists and their work experience influence the solutions that they use in practice as part of onboarding. HR specialists over 25 understand to a greater extent than their younger colleagues how important it is for new employees to know about the values, mission, and vision of the company in which they take up employment and emphasize the transfer of such knowledge as part of onboarding to newly hired staff. In addition, the seniority of HR specialists is one of the factors influencing the choice of onboarding solutions, and so is gender. Female HR specialists attach more importance to giving employees the necessary access in the new workplace (such as a parking space, entry card, e-mail address, etc.) than male HR specialists. The obtained research results, apart from the cognitive value, indicate the possibility of using them in the practice of management, e.g., when employing specialists in the area of HR.

The study was conducted in compliance with research ethics. The research problems addressed in the study accounted for ethical concerns, both of a social and individual nature, e.g., in relation to the subject matter under analysis and the way in which questions were formulated and presented to the respondents. Research procedures described by other researchers [88] were similarly addressed. The participants of the study were informed of the aims of the study and the research tool used in it [92]. All of the respondents were aware that they could opt out of the study any time. Furthermore, the requirements of respondents’ anonymity and the security of their personal data were met. The study in no way compromised the sense of freedom of any of the respondents [93].

The study described in the article is not devoid of limitations. These include purposive sampling and the small sample size. Moreover, the comparison of different types of companies would benefit from having access to the same number of companies of each type. Further validation of the research tool used in the study is also recommended. 

When considering directions for further research, it would be advisable to plan a mixed research paradigm: more extensive quantitative studies followed by in-depth qualitative studies, focused not on types of companies but on particular groups of employees, diversified with reference to their age, seniority, and position. Moreover, in the authors’ opinion, future studies should also include HR specialists and employees of other nationalities, which would allow for comparative analyses of the preferences of Polish HR specialists and employees and the preferences of HR specialists and employees from other countries and cultures. An interesting direction of future research may also include obtaining opinions of employees and managers on onboarding separately—both in quantitative and qualitative studies—and identifying which onboarding practices these two groups find the most effective and why.

In addition, further directions of research should include more in-depth analyses of onboarding practices in various types of enterprises: service (different types, e.g., IT sector, consulting services sector, or delivery companies), manufacturing, and commercial enterprises separately. In the case of further research, it could also be interesting to analyze the effects of onboarding practices in specific enterprises and to indicate whether the onboarding solutions used affect the greater integration of employees with the company, their job satisfaction, and staying in the company.

In addition, bearing in mind the findings of the study conducted by Danvil-del-Valle et al. [94] that organizations are more willing to implement innovation when the introduction of good practices related to the onboarding process takes place early in their development cycle rather than those in which this process is introduced later, it would be worthwhile to also focus attention in future studies on the time factor in a company’s operation in the market as a variable that influences its HR practices and solutions implemented in the field of HR, including onboarding. However, it should be emphasized that, regardless of its limitations, the study described in the article has revealed a rather optimistic picture of the implementation of onboarding practices in the analyzed Polish companies and its perception by HR specialists who are responsible for this process in practice.

## Figures and Tables

**Table 1:** The type of company and the type of onboarding implemented.

Type of Company		General	Team	Position
Manufacturing	Mean	4,0800	1,8400	2,5600
	N	25	25	25
	Standard deviation	1,32035	0.37417	0.86987
Trading	Mean	4,1471	1,7353	2,5882
	N	34	34	34
	Standard deviation	1,15817	0.61835	0.95719
Service	Mean	4,0588	1,7983	2,6050
	N	119	119	119
	Standard deviation	1,36104	0.53011	0.69158
Total	Mean	4,0787	1,7921	2,5955
	N	178	178	178
	Standard deviation	1,31248	0.52781	0.76975

Source: own elaboration.

**Table 2:** Preference for a particular type of onboarding among women and men.

Gender		1 *	2	3	4	5	6	7	8	9	10
Man	Mean	4,38	4,0000	3,8537	3,5714	4,0238	4,0000	4,1875	3,6053	3,9130	3,8043
	N	42	43	41	35	42	46	48	38	46	46
	Standard deviation	1,147	1,17514	1,25620	1,37810	1,27811	1,36626	1,29904	1,30569	1,29660	1,34362
Woman	Mean	4,50	4,0777	4,2970	3,9278	3,9035	4,0734	4,1810	3,8384	3,9832	4,0088
	N	108	103	101	97	114	109	116	99	119	114
	Standard deviation	1,164	1,28100	1,14494	1,31694	1,38876	1,31026	1,29614	1,38289	1,30827	1,30686
Total	Mean	4,47	4,0548	4,1690	3,8333	3,9359	4,0516	4,1829	3,7737	3,9636	3,9500
	N	150	146	142	132	156	155	164	137	165	160
	Standard deviation	1,157	1,24724	1,19081	1,33746	1,35684	1,32309	1,29300	1,36119	1,30146	1,31656

Source: own elaboration. * Legend: 1—A referral to pre-employment medical examination, 2—Handing tools/equipment necessary for performing the job (e.g., laptop, phone, clothing), 3—Granting access to places and services necessary for performing the job (e.g., parking lot, employee ID card, email address), 4—Providing information on the company’s values, vision, and mission, 5—Ensuring that employee-hiring formalities are completed properly, 6—Introducing employees to their superiors, 7—Introducing employees to their department/team, 8—Providing information on the departments or organizational units that are the most important from the point of view of an employee’s position, 9—Providing information on an employee’s duties and ways of performing them, 10—Providing information on the company’s policies, procedures, and relations.

**Table 3:** Preference for a particular type of onboarding and the respondents’ gender.

Type of Statistics Used	1 *	2	3	4	5	6	7	8	9	10
Mann–Whitney’s U	2083,000	2057,000	1595,500	1424,000	2286,000	2440.000	2783,000	1663,000	2613,500	2361,500
Wilcoxon’s W	2986,000	3003,000	2456,500	2054,000	8841,000	3521,000	3959,000	2404,000	3694,500	3442,500
Z	−1,069	−0.737	−2,384	−1,492	−0.466	−0.290	−0.004	−1,104	−0.483	−1,059
Asymptotic significance (2-sided)	0.285	0.461	0.017	0.136	0.641	0.772	0.997	0.270	0.629	0.289

Source: own elaboration; * The legend of the table has been placed below Table 2.

**Table 4:** Preference for a particular type of onboarding among respondents at a given age range.

Age		1 *	2	3	4	5	6	7	8	9	10
Under 25	Mean	4,50	4,5385	4,1667	3,8500	3,8889	4,2000	4,3333	3,8462	4,1786	4,1538
	N	22	26	30	20	27	25	27	26	28	26
	Standard deviation	1,185	0.81146	0.94989	1,30888	1,08604	1,08012	1,00000	1,18970	0.90487	0.92487
25–30	Mean	4,58	4,0000	4,5417	4,2400	4,3704	4,2308	4,5556	4,1250	4,1538	4,1852
	N	24	26	24	25	27	26	27	24	26	27
	Standard deviation	0.929	1,26491	0.88363	1,12842	1,07946	1,10662	1,01274	0.99181	1,18970	1,03912
31–35	Mean	4,64	3,6364	4,0000	4,1000	3,7500	4,0000	4,0833	3,4545	3,9091	4,5000
	N	11	11	11	10	12	11	12	11	11	10
	Standard deviation	0.924	1,43337	1,48324	1,10050	1,48477	1,41421	1,37895	1,57249	1,57826	1,08012
36–40	Mean	4,47	3,8333	4,3333	4,3333	4,1176	4,1875	4,0556	3,8182	3,8947	3,7895
	N	19	18	15	15	17	16	18	11	19	19
	Standard deviation	1,264	1,72354	1,29099	1,29099	1,45269	1,32759	1,47418	1,53741	1,62941	1,58391
41–45	Mean	4,39	4,0154	4,0161	3,5000	3,7808	3,9221	4,0500	3,6615	3,8519	3,7692
	N	74	65	62	62	73	77	80	65	81	78
	Standard deviation	1,237	1,17915	1,31189	1,41131	1,48368	1,45788	1,40433	1,48194	1,34268	1,44979
Total	Mean	4,47	4,0548	4,1690	3,8333	3,9359	4,0516	4,1829	3,7737	3,9636	3,9500
	N	150	146	142	132	156	155	164	137	165	160
	Standard deviation	1,157	1,24724	1,19081	1,33746	1,35684	1,32309	1,29300	1,36119	1,30146	1,31656

Source: own elaboration; * The legend of the table has been placed below the Table 2.

**Table 5:** Preference for a particular type of onboarding and the respondents’ age (tested value a, b).

Type of Statistics Used	1 *	2	3	4	5	6	7	8	9	10
Kruskal–Wallis’ H	0.499	5,312	5,258	10.079	5,242	1,162	4,104	1,603	1,790	4,122
Df	4	4	4	4	4	4	4	4	4	4
Asymptotic significance	0.974	0.257	0.262	0.039	0.263	0.884	0.392	0.808	0.774	0.390

Source: own elaboration; * The legend of the table has been placed below the Table 2. a. Kruskal–Wallis test; b. Grouping variable: Age.

**Table 6:** Preference for a particular type of onboarding among HR specialists with given seniority.

Seniority	Statistics	1 *	2	3	4	5	6	7	8	9	10
Under 9 months	Mean	4,33	4,3684	4,1905	4,0000	4,0455	4,1579	4,2857	3,5500	4,0952	4,1053
	N	15	19	21	17	22	19	21	20	21	19
	Standard deviation	1,397	0.89508	1,03049	1,22474	0.95005	1,16729	1,00712	1,39454	1,17918	1,04853
9 months–2 years	Mean	4,83	4,1667	4,1600	3,7500	3,9545	4,3333	4,4583	4,1304	3,9583	4,1250
	N	18	24	25	20	22	24	24	23	24	24
	Standard deviation	0.383	1,09014	0.89815	1,33278	1,17422	0.96309	0.93153	1,05763	0.99909	0.99181
2–6 years	Mean	4,77	4,2564	4,5750	4,2286	4,2683	4,3000	4,4889	3,7429	4,3721	4,3095
	N	43	39	40	35	41	40	45	35	43	42
	Standard deviation	0.751	1,16343	0.90263	1,11370	1,24548	1,15913	1,10005	1,29121	0.97647	1,02382
Over 6 years	Mean	4,23	3,7969	3,8750	3,5833	3,7042	3,7917	3,8784	3,7288	3,7013	3,6533
	N	74	64	56	60	71	72	74	59	77	75
	Standard deviation	1,360	1,40498	1,45305	1,45313	1,54360	1,50994	1,50754	1,49517	1,51367	1,54652
Total	Mean	4,47	4,0548	4,1690	3,8333	3,9359	4,0516	4,1829	3,7737	3,9636	3,9500
	N	150	146	142	132	156	155	164	137	165	160
	Standard deviation	1,157	1,24724	1,19081	1,33746	1,35684	1,32309	1,29300	1,36119	1,30146	1,31656

Source: own elaboration; * The legend of the table has been placed below Table 2.

**Table 7:** Preference for a particular type of onboarding among the respondents with given seniority (tested value a, b).

Tested Value ^a,b^										
Type of Statistics Used	1 *	2	3	4	5	6	7	8	9	10
Kruskal–Wallis’ H	5,870	4,443	8,631	5,275	4,736	3,903	7,895	1,950	6,061	5,420
Df	3	3	3	3	3	3	3	3	3	3
Asymptotic significance	0.118	0.217	0.035	0.153	0.192	0.272	0.048	0.583	0.109	0.143

Source: own elaboration; * The legend of the table has been placed below the Table 2. ^a^. Kruskal–Wallis test; ^b^. Grouping variable—Work experience—seniority.

**Table 8:** The type of company and assignment of a buddy (contingency table).

Presence of Buddy	Manufacturing Company		Trading Company		Service Company		Total
Answers	N	%	N	%	N	%	N (%)
Yes	20	80	17	50	58	48.7	95 (53.4)
No	5	20	17	50	61	51.3	83 (46.6)
Total	25	100	34	100	119	100	178 (100)

Source: own elaboration.

**Table 9:** The type of company and assignment of a buddy (Pearson’s chi-squared test).

Type of Statistics Used	Value	df	Asymptotic Significance (2-Sided)
Pearson’s chi-squared test	8,304	2	0.016
Odds ratio	8,903	2	0.012
Linear correlation test	6,270	1	0.012

Source: own elaboration.

## Data Availability

The data presented in this study are available on request from the corresponding author.
